# Ubiquitin-specific protease 11 structure in complex with an engineered substrate mimetic reveals a molecular feature for deubiquitination selectivity

**DOI:** 10.1016/j.jbc.2023.105300

**Published:** 2023-09-28

**Authors:** Sigrun K. Maurer, Matthias P. Mayer, Stephanie J. Ward, Sana Boudjema, Mohamed Halawa, Jiatong Zhang, Simon G. Caulton, Jonas Emsley, Ingrid Dreveny

**Affiliations:** Biodiscovery Institute, School of Pharmacy, University of Nottingham, Nottingham, United Kingdom

**Keywords:** ubiquitin-specific protease, crystal structure, cysteine protease, deubiquitylation (deubiquitination), protease, selectivity, ubiquitin, deubiquitinase, fusion tag

## Abstract

Ubiquitin-specific proteases (USPs) are crucial for controlling cellular proteostasis and signaling pathways but how deubiquitination is selective remains poorly understood, in particular between paralogues. Here, we developed a fusion tag method by mining the Protein Data Bank and trapped USP11, a key regulator of DNA double-strand break repair, in complex with a novel engineered substrate mimetic. Together, this enabled structure determination of USP11 as a Michaelis-like complex that revealed key S1 and S1′ binding site interactions with a substrate. Combined mutational, enzymatic, and binding experiments identified Met^77^ in linear diubiquitin as a significant residue that leads to substrate discrimination. We identified an aspartate “gatekeeper” residue in the S1′ site of USP11 as a contributing feature for discriminating against linear diubiquitin. When mutated to a glycine, the corresponding residue in paralog USP15, USP11 acquired elevated activity toward linear diubiquitin in-gel shift assays, but not controls. The reverse mutation in USP15 confirmed that this position confers paralog-specific differences impacting diubiquitin cleavage rates. The results advance our understanding of the molecular basis for the higher selectivity of USP11 compared to USP15 and may aid targeted inhibitor development. Moreover, the reported carrier-based crystallization strategy may be applicable to other challenging targets.

Ubiquitin-specific proteases (USPs) are an integral part of the enzymatic network that regulates key cellular events by altering the ubiquitination status of a wide range of proteins (1). Substrates for deubiquitination have a “distal ubiquitin” moiety in common that is typically conjugated to a target’s lysine residue and binds to the protease extensive S1 binding pocket. This leaves the challenge of ubiquitin conjugated target discrimination to other regions of these proteases. A key region for target interaction is the S1′ site, which however appears shallow in the canonical USP fold (Fig. S1). How specific substrates are selected by each of the 56 USP enzymes encoded in the human genome remains an important question in the field and is a major factor for inhibitor development.

USP11 regulates multiple important cellular functions, including DNA double-strand break repair, cell cycle progression (2, 3, 4, 5), and signaling pathways such as transforming growth factor beta signaling (6, 7). Moreover, USP11 is involved in viral ribonucleic acid replication (8) and is dysregulated in different types of cancer including breast, ovarian, pancreatic cancer, melanoma, and myeloid leukemia. USP11 inhibition is a promising treatment strategy in synthetic lethality approaches (9, 10, 11, 12, 13, 14), yet at present structural information on USP11 as a platform for rational inhibitor design is lacking.

USP11 has two distantly related paralogs, USP4 and USP15, which display higher sequence identity with each other (56.9% over the entire sequence) than USP11 (41.4% shared sequence identity with both, USP4 and USP15). The paralogs share the same modular structure consisting of an N-terminal domain present in USPs and a ubiquitin-like domain (15, 16, 17), followed by the USP protease domain composed of subdomains D1 and D2 interspersed by an insertion (Fig. 1*A*). In contrast to USP11, catalytic domain structures of USP4-D1D2 (18) and USP15-D1D2 (19, 20) in the free form or bound to an inhibitor are available but the molecular basis of substrate interactions is currently unknown. While these paralogs function in related or common pathways, USP11, USP4, and USP15 show significant differences in regulation and substrate specificity (15, 18, 21). For example, ancillary domains do not significantly modulate the catalytic activity of USP11 or USP15 using ubiquitin-7-amino-4-methylcoumarin or diubiquitin substrates, but in USP4 they facilitate ubiquitin discharge (15, 18). USPs are generally promiscuous in cleaving ubiquitin chains (1, 22). However, for USP11, a preference for Lys^63^-, Lys^6^-, Lys^33^-, and Lys^11^-linked over Lys^27^-, Lys^29^-, Lys^48^-linked poly-ubiquitin chains has been observed *in vitro* consistent with its role in DNA damage repair. Linear ubiquitin chains are particularly poor substrates for USP11 (15, 22, 23). In contrast, USP15 readily accepts different types of ubiquitin chains (24) and a broader range of substrates (25). How this paralog-specific selectivity is achieved is currently unknown.

**Figure 1:**
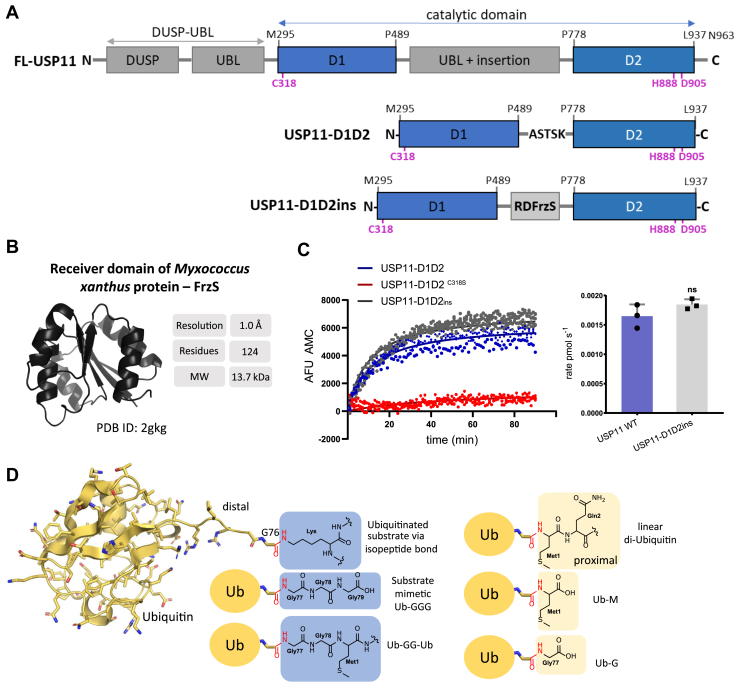
**Design of USP11 catalytic domain and ubiquitin substrate constructs**. *A*, *top:* schematic representation of the hUSP11 domain structure (UniProt P51784). The catalytic subdomains D1 and D2 are depicted in *blue* and the catalytic triad residues marked in *magenta*. Additional domains are shown in *gray*. *Below:* schematic representation of the USP11-D1D2 (residues Met^295^-Leu^937^) construct with the insertion replaced by an ASTSK linker or the RDFrzS loop insertion tag shown in *light gray* to engineer USP11-D1D2_ins_. *B,* receiver domain structure from the *Myxococcus xanthus* protein FrzS (26) used as an insertion tag. *C*, progress curves of USP11-D1D2 ubiquitin-7-amino-4-methylcoumarin cleavage in comparison with USP11-D1D2 ^C318S^ and USP11-D1D2_ins_ constructs with a bar chart (unpaired two-tailed *t* test; n = 3 independent experiments; error = SD) highlighting that the insertion does not significantly affect the catalytic activity. *D*, ubiquitin C-terminal tail modified substrates as used in this study with respective tail modifications shown as chemical structures. USP, ubiquitin-specific protease.

Here, we present the first structure of the USP11 catalytic domain and discern differences with paralogs USP4 and USP15. The structure of USP11 trapped with an engineered USP substrate revealed critical binding interactions and led to the identification of an unexpected structural feature that contributes to USP11’s substrate selectivity. We also introduce the RDFrzS domain as a novel fusion tag that can be incorporated into loop regions to improve crystallization outcomes.

## Results

### A novel loop insertion tag strategy utilized for the crystallization of USP11

Previous strategies for replacing an insert region containing significant disorder to crystallize USP11 generated the USP11-D1D2 construct (Fig. 1*A*) (15, 19), but did not yield any diffraction quality crystals. Therefore, a novel insert–substitution strategy was developed. Using search parameters such as size, availability of a high-resolution structure (<1.6 Å), and expression yield, we mined the Protein Data Bank (PDB) and identified the N-terminal 13.7 kDa receiver domain (RD) of bacterial *Myxococcus xanthus* social motility protein FrzS (26) as a potential candidate tag for carrier-driven crystallization. The RDFrzS structure (PDB ID: 2gkg) was determined to an atomic resolution of 1 Å and adopts a globular fold consisting of a central β-sheet surrounded by five α-helices (Fig. 1*B*). Moreover, the RDFrzS protein can be highly concentrated and has been crystallized in multiple different space groups, indicating high solubility and crystallizability (26). We reasoned that due to the proximity of its N- and C-terminal Cα atoms (∼6.3 Å), RDFrzS can be inserted to replace predicted flexible loop regions or insertions in target proteins, such as USP11. Two surface entropy reducing (SER) modifications to the RDFrzS sequence were also incorporated. The final construct, schematically depicted in Figure 1*A* consisted of the USP11 catalytic core (residues Met^295^-Pro^489^ and Pro^778^-Leu^937^), with the two subdomains linked by the novel RDFrzS fusion tag, which substitutes the 289-aa long insertion between Pro^489^ and Pro^778^ (USP11-D1D2_ins_). Measurements of the catalytic activity of USP11-D1D2_ins_ compared to the original catalytic core protein USP11-D1D2 (15) did not show any significant differences, confirming that the catalytic function of USP11-D1D2_ins_ is equivalent to USP11-D1D2 (Fig. 1*C*).

### USP11 trapping with an engineered substrate mimetic

To gain insights into substrate recognition, we engineered a novel substrate mimetic comprising ubiquitin extended by three C-terminal glycine residues. The design principle underlying the ubiquitin-triple-gly (Ub-GGG) substrate mimetic in comparison with other substrates is illustrated in Figure 1*D*. Ub-GGG represents a close substrate mimetic of ubiquitin conjugated to a substrate’s lysine *via* an isopeptide bond as this extension can adopt the extended shape of a lysine side chain and like an isopeptide bond harbors a CH_2_ group right next to the scissile bond. To trap a Michaelis complex intermediate for crystallization studies, we mutated the USP11 active site cysteine and confirmed that the mutant was inactive (Fig. 1*C*). USP11-D1D2_ins_^C318S^ was coexpressed with Ub-GGG and complex formation was confirmed by gel filtration (data not shown). The complex was crystallized using sparse matrix crystallization screening, and the largest crystals were obtained in 100 mM Tris-Bicine pH 8.5, 30 mM sodium nitrate, 30 mM sodium phosphate dibasic, 30 mM ammonium sulphate, 11.25% (v/v) MPD; 11.25% (v/v) PEG 1000; 11.25% (w/v) PEG 3350 with 5 mM cadmium chloride after extensive optimizations. Crystals belonged to space group P 2_1_ 2_1_ 2 and diffracted to 2.44 Å resolution.

### Structure of the USP11–Ub-GGG substrate complex

**Figure 2:**
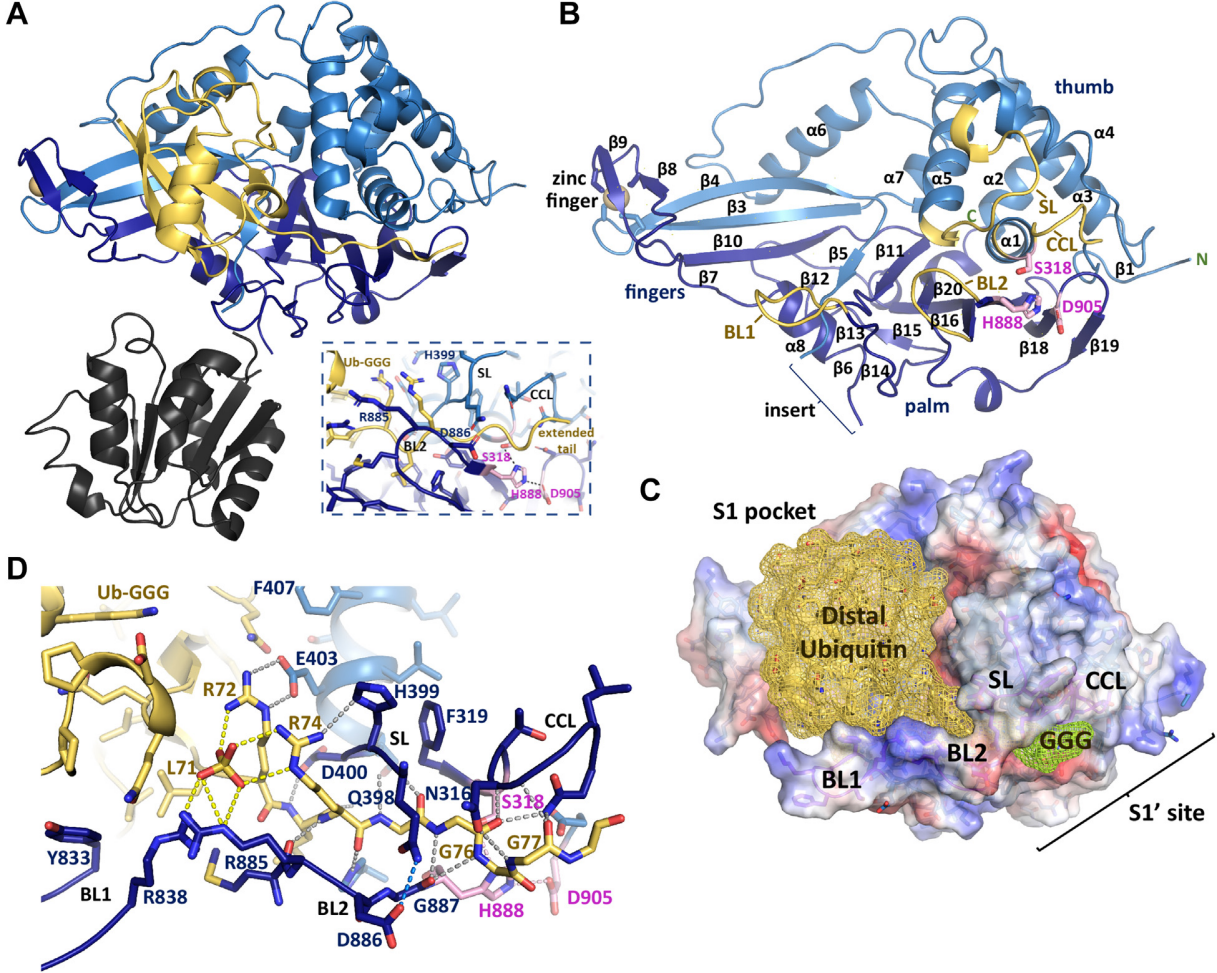
**USP11 crystal structure in complex with substrate Ub-GGG**. *A*, crystal structure of USP11-D1D2_ins_ with the USP11 catalytic core depicted in *blue*, Ub-GGG in *yellow*, and the RDFrzS insertion in *dark gray*. The *yellow sphere* represents the cadmium ion present in the structure. Inset shows a zoomed in view of the extended substrate tail region. *B*, cartoon representation of USP11-D1D2 catalytic core without Ub-GGG and the insertion shown, with the active site loops and secondary structure elements labeled; *light blue* D1, *dark blue* D2, catalytic triad residues *pink in stick representation*, and the BL1 (blocking loop 1), BL2 (blocking loop 2), SL (switching loop), and catalytic cleft loop (CCL) are labeled in *dark yellow*. *C*, USP11-D1D2 surface representation colored according to the electrostatic potential with Ub-GGG in *yellow mesh representation*. The extended glycine tail residues are colored in *lime* and the active site loop and S1, S1′ regions are indicated. *D*, close-up view of the Ub-GGG tail–binding channel showing key interactions. Likely H-bonding interactions are shown as *black dashed lines* and key residues are labeled. USP, ubiquitin-specific protease; Ub-GGG, ubiquitin-triple-gly.

The structure of the USP11 catalytic core domain in complex with Ub-GGG was solved by molecular replacement. Data collection and refinement statistics are shown in Table 1. Both copies of USP11 in the asymmetric unit (ASU) superimpose well with differences seen in the position of the inserted crystallization tag RDFrzS (Fig. S2*A*). The RDFrzS tag is involved in several crystal contacts (Fig. S2*B*). USP11-D1D2 adopts the canonical USP protease fold with thumb, palm, and finger regions (Fig. 2*A*). The zinc finger (Cys^468^, Cys^471^, Cys^802^, Cys^805^) coordinates a cadmium ion, due to the crystallization additive CdCl_2_. The catalytic triad residues C318S, His^888^, and Asp^905^ are within hydrogen-bonding distance and adopt an active conformation (Fig. 2*B*). Electron density maps show both copies of the USP11 catalytic core interact with Ub-GGG in the S1 binding site with clear electron density for the entire extended glycine tail observed (Fig. S3). The buried surface area formed comprises ∼ 2286 Å^2^, including ∼46 H-bonding interactions and ten salt bridges according to PISA (27). The active site loop regions, namely catalytic cleft loop (CCL, T^312^-NLGNTS-F^319^), switching loop (SL, S^391^-QFLGYQQHDS-Q^402^), blocking loop 1 (BL1, S^832^-YTKFS-R^838^), and blocking loop 2 (BL2, G^882^-GMR-D^886^) engage in Ub-GGG substrate interactions and are well ordered (Fig. 2*B*).

**Table:** Data collection and refinement statistics

USP11-D1D2_ins_^C318S^ in complex with Ub-GGG	
Data collection	
Space group	P 2_1_ 2_1_ 2
Cell dimensions	
*a*, *b*, *c*	94.32 Å, 186.10 Å, 75.76 Å
Resolution (outer shell)	48.0–2.44 Å (2.52–2.44)
*R*_*merge*_ (outer shell)^a^	0.40 (2.66)
*R*_*pim*_ (outer shell)^b^	0.114 (0.782)
*I*/σ*I* (outer shell)	7.6 (1.45)
CC_1/2_ (outer shell)^c^	0.988 (0.437)
Completeness (outer shell)	99.8 (98.3) %
Redundancy (outer shell)	13.4 (13.3)
Total Reflections (outer shell)	675,489 (60,034)
Wilson B-factor	33.7 Å^2^
Refinement	
Resolution range	48.0–2.44 Å
No. of unique reflections	50,405 (4955)
*R*_work_/*R*_free_^d^	0.173/0.230
No. atoms	
Protein	8850
Other	42
Water	505
*B*-factors (Å^2^)	
Protein	44.3
Ligand	49.4
Water	39.9
Ramachandran	
Favoured regions (%)	96.85
Allowed regions (%)	2.78
Outliers (%)	0.37
R.m.s. deviations	
Bond lengths (Å)	0.0044
Bond angles (°)	0.81

a R_merge_ = Ʃ_h_ Ʃ_i_ | *I*_*i(h)*_ - *Ī*_*(h)*_ |/Ʃ_h_ Ʃ_i_*I*_*i(h)*_.

b R_pim_= Ʃ_h_ (1/N-1)^1/2^ Ʃ_i_ | *I*_*i(h)*_ - *Ī*_*(h)*_ |/Ʃ_h_ Ʃ_i_*I*_*i(h)*,_*h* is the given reflection, *Ī*_*(h)*_ is the average intensity of each reflection, and i is the ith measurement of reflection *h*.

c CC_1/2_ is the Pearson correlation coefficient between random half-datasets.

d R_work_ = Ʃ_h_ | *F*_*obs(h)*_ – *F*_*calc(h)*_ |/Ʃ_h_*F*_*obs(h)*_; R_free_ corresponds to the R_work_ based on 5% of the data excluded from refinement.

The substrate-binding channel is in a closed conformation. Several noticeable features characterize the engagement of USP11 with the substrate’s C-terminal tail (Fig. 2, *C* and *D*). The Ub-GGG NH group of Gly^77^ that is equivalent to a lysine side chain’s NH group in an isopeptide bond forms a hydrogen-bonding interaction with USP11 Gly^887^ from the BL2 region. The Gly^77^ CH_2_ group (equivalent to a lysine’s side chain Cε) engages in van der Waals contacts with the catalytic histidine. The C-terminal Gly^78^ and Gly^79^ of the triple-gly extension, display higher B-factors and extend outward from the catalytic core in slightly different orientations in the two copies of the ASU (Fig. S2*A*). USP11 Asn^313^ in the CCL region interacts with the carbonyl group of the Ub-GGG Gly^76^ and USP11 Asn^316^ forms a hydrogen-bonding interaction with Ub-GGG Gly^78^. The USP11 SL loop Gln^398^ side chain closes over the substrate’s C-terminal tail by forming hydrogen-bonding interactions with the Asp^886^ side chain and Gly^887^ from the BL2 region (Fig. 2*D*). USP11 Phe^831^ is part of a hydrophobic region that accommodates Ub-GGG Val^70^, Leu^71^, and Leu^73^. Ub-GGG Arg^72^ forms an electrostatic interaction with USP11 Glu^403^ in helix α5. Density consistent with a phosphate ion (from the crystallization mother liquor) forms part of the interface between USP11 and the substrate’s C-terminal tail and the presence of 10 mM phosphate slightly increased the reaction rate (Fig. S4). In the USP11 BL1 region, only Tyr^833^ and Arg^838^ interact with the substrate through hydrophobic interactions with Ile^36^ and hydrogen-bonding interactions with Gln^40^, respectively. Together, the structure reveals key molecular features of the Michaelis complex intermediate by capturing the interactions of USP11 with a trapped, engineered USP substrate.

### Structural differences between USP11 and the paralogs USP4 and USP15

**Figure 3:**
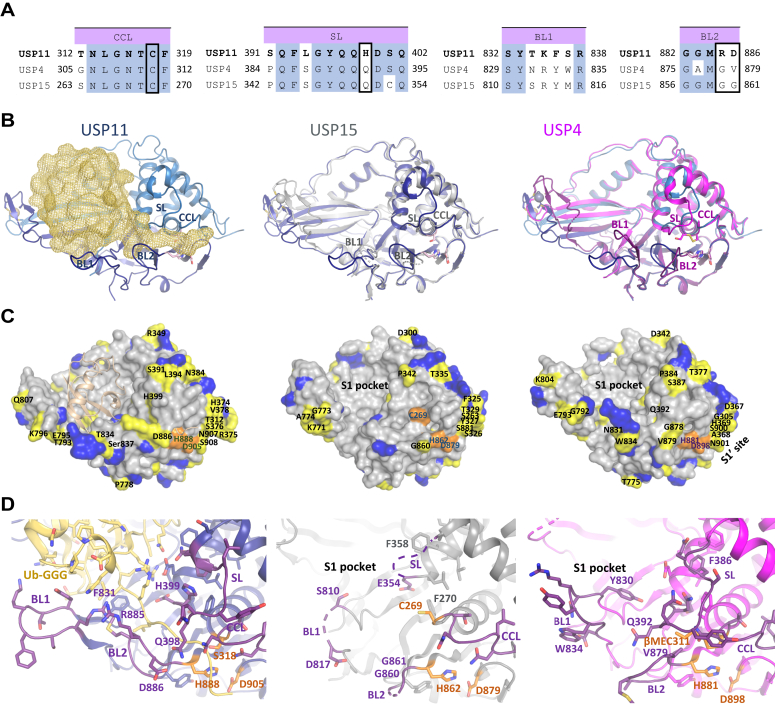
**Comparison of paralogues USP11, USP4, and USP15.***A*, sequence alignments of hUSP11 (UniProt P51784), hUSP4 (UniProt Q13107), and hUSP15 (Q9Y4E8-2) active site loop regions: catalytic cleft loop (CCL), switching loop (SL), blocking loop 1 (BL1), and blocking loop2 (BL2). *Blue shading* denotes identical residues between the paralogs and *boxed residues* highlight the basis for generated mutants in Figure 4. *B*, comparison of the USP11 catalytic core (*blue*) in complex with Ub-GGG (*left*, *yellow mesh*) superimposed onto USP15 (*middle, gray*, PDB: 6gha) and USP4 structures (*right, magenta,* PDB: 2y6e) in cartoon representation with active site loops labeled. *C*, comparison of USP11, USP15, and USP4 catalytic core structures in surface representation in the same order as in *B* highlighting sequence conservation: *gray residues* are identical between all paralogs, *yellow residues* are different in USP11 compared to USP4 and USP15, and *blue residues* have similar properties in USP11 compared to USP4 and USP15. The catalytic triad residues are shown in *orange*. *D*, active site region of USP11 (*blue*) in complex with Ub-GGG (*yellow*) shown in the same orientation as USP15 (*gray, middle*) and USP4 (*magenta, right*) highlighting the different conformations of the loop regions depicted in *dark purple*, catalytic triad residues in *orange*, and additional residues of interest are labeled. USP, ubiquitin-specific protease; Ub-GGG, ubiquitin-triple-gly.

The USP11 catalytic core shares ∼66% sequence identity with the paralogs USP4 and USP15, respectively. Structures of USP15 in the free state or bound to either a small molecule or truncated and modified ubiquitin variant inhibitors (19, 20) and USP4 as a β-mercaptoethanol–bound adduct (18) have been solved. No USP4 or USP15 structure in complex with a reaction product or substrate has been reported to date. Hence, the following comparisons reflect differences between paralogs as well as the substrate-bound *versus* free state. The structure of USP11 in complex with the substrate Ub-GGG can be superimposed with USP4 (PDB ID: 2y6e) with a RMSD of 0.7 Å (over 1791 atoms out of 2735) and with USP15 in the free state (PDB ID: 6gha) with an RMSD of 0.7 Å (over 1719 atoms out of 2603), respectively. The active site CCL and SL loop regions share high-sequence conservation between the three paralogs, whereas the BL1 and BL2 loop regions are less well conserved (Figs. 3*A* and S5).

#### Catalytic cleft, BL1 and 2, and SL regions

The CCL regions in the structures of USP11 (Thr^312^-Phe^319^) and USP4 (Gly^305^-Phe^312^) adopt largely similar conformations. The first turn of helix α1 harbors the catalytic cysteine that is in hydrogen-bonding distance with the other catalytic triad residues. In the USP15 free state, residues Asn^267^-Phe^270^ including the active site cysteine adopt a catalytically incompetent conformation (Fig. 3, *B*–*D*). In contrast, the catalytic triad histidine and aspartate closely superimpose in all available paralog structures (USP11 His^888^ and Asp^446^, USP4 His^881^ and Asp^898^, USP15 His^862^ and Asp^879^). The SL regions (USP11 Ser^391^-Gln^402^, USP4 Pro^384^-Gln^395^, USP15 Pro^342^-Gln^353^) adopt different conformations, likely attributable to the presence and absence of ubiquitin (Fig. 3*D*). USP11 harbours a QHD sequence in the SL loop (residues 398–400) that interacts with the substrate as opposed to the more typical “QQD” box in USP4 and USP15. The USP11 SL His^399^ side chain interacts with Ub-GGG Arg^74^. BL1 loop conformations also differ associated with the substrate-binding status (Fig. 3, *B* and *D*): USP11 Phe^831^ interacts with Ub-GGG Leu^71^ and Leu^73^. In the absence of a substrate’s distal ubiquitin moiety equivalent residues in USP4 (Phe^828^) and USP15 (Phe^809^) block the Leu^73^ binding pocket. The BL1 loop in the USP11 complex structure is rearranged to accommodate the substrate, whereby Tyr^833^ and Arg^838^ form most of the contacts with Ub-GGG (Ile^36^, Gln^40^, Leu^71^, Arg^72^). In the USP4 structure, Tyr^830^ blocks the substrate C-terminal tail binding channel by forming a hydrogen-bonding interaction with Asp^393^ in the SL. In USP15, large parts of the BL1 are flexible in the free state. The BL2 region is one of the least conserved active site loops (USP11 Tyr^881^-Gly^887^, USP4 Tyr^874^-Gly^880^, USP15 Tyr^855^-Gly^861^) and adopts different conformations in the structures. USP11 residue Arg^885^ (Fig. S4) is not conserved in either USP4 (Gly^878^) or USP15 (Gly^859^). In addition, USP11 Asp^886^ (USP4 Val^879^ and USP15 Gly^860^) contributes to BL2 differences by interacting with Gln^398^ in the SL loop (Fig. 3*D*).

#### USP11 S1 and S1′ pockets

In general, residues in the S1 distal ubiquitin binding pocket are relatively well conserved. The S1′ binding region that engages either a proximal Ub moiety in a polyubiquitin chain or different substrates linked to the C terminus of Ub displays considerable differences (Figs. 3*C* and S5).

At the rim of the S1 pocket, helix α4 is shifted outward to accommodate the Ub-GGG substrate in USP11 compared to the free paralog structures. USP11 Tyr^424^ and Glu^426^ between helices α5 and α6 at the top of the S1 pocket interact with Ub-GGG. At the bottom of the S1 binding pocket, USP11 Phe^462^ and Leu^484^ face Ub-GGG His^68^ and show flipped conformations compared to USP4 (Phe^455^, Leu^477^) and USP15 (Phe^413^, Leu^435^). Furthermore, in USP11 Cys^482^ and Tyr^483^ interact with the Ile^44^ patch of Ub-GGG. The finger regions in USP11 (Lys^463^-Thr^477^ and Thr^789^-Leu^817^) accommodate the distal ubiquitin core of the substrate. This “open hand” conformation sees shifts of approximately 9 Å compared to equivalent Cα positions in USP15 and USP4, which adopt a “closed hand” conformation in the absence of a substrate. The target substrate–specific S1′ site is characterized by low sequence conservation between the paralogs (Fig. 3*C*). Nevertheless, USP11 main chain residues His^373^-Ser^376^ superimpose well onto USP4 Arg^366^-His^369^ and USP15 Lys^324^-Tyr^327^. Furthermore, several other differences occur in the S1′ region as indicated in Figure 3.

### Paralog-specific differences in product and substrate-binding parameters

**Figure 4:**
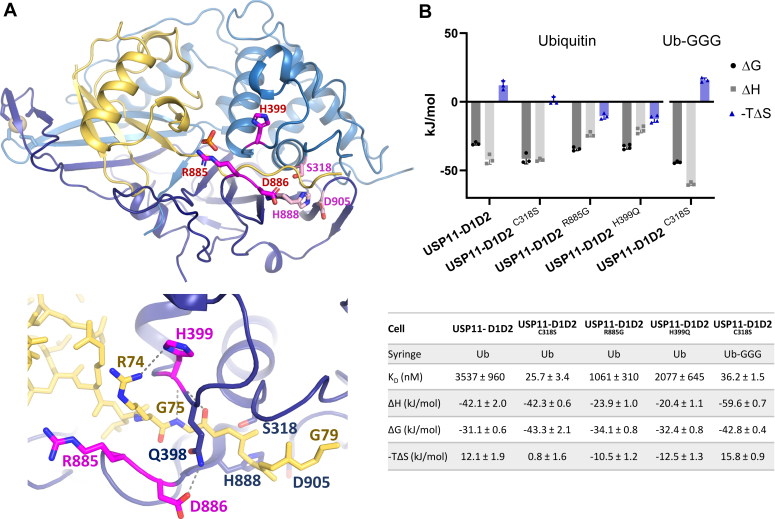
**USP11 interactions with product ubiquitin and impact of mutations.***A*, structure of USP11 in complex with the Ub-GGG substrate with residues subjected to mutagenesis to probe paralog-specific differences shown in *magenta stick representation* and a zoom in view below highlighting putative H-bonding interactions. *B*, thermodynamic parameters from ITC binding assays for USP11-D1D2 or indicated USP11-D1D2 mutants with mono-ubiquitin (product) or Ub-GGG, in graphical (error = SD) and table (n = 3–4 independent experiments; error = SEM) format. USP, ubiquitin-specific protease; Ub-GGG, ubiquitin-triple-gly.

To further investigate paralog-specific differences, we utilized site-directed mutagenesis coupled with isothermal titration calorimetry (ITC) and focused on differences in the catalytic site loop regions (Fig. 3*A*). Notably, in USP11 residues His^399^ (SL) as well as Arg^885^ and Asp^886^ (BL2) are not conserved in USP4 and USP15. To investigate these differences, we generated “chimeras” introducing USP15/USP4 or USP15-like mutations into USP11 (Figs. 3*A* and 4*A*).

**Figure 5:**
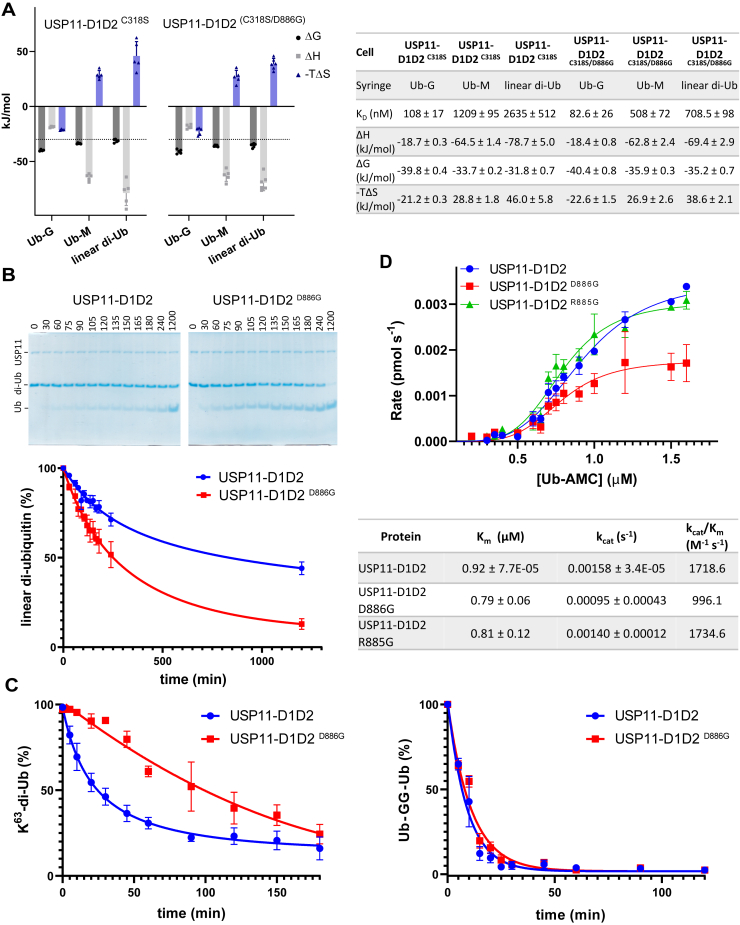
**Impact of paralog-specific differences in the BL2 region on USP11 substrate binding and cleavage.***A*, thermodynamic parameters from ITC binding assays for USP11-D1D2 ^C318S^ and USP11-D1D2 ^(C318S/D886G)^ interactions with Ub-G (minimal substrate), ubiquitin-methionine (Ub-M, truncated linear di-Ub substrate), and linear diubiquitin (poor substrate), in graphical (error = SD) and tabulated format (error = SEM; n ≥ 4). *B*, representative gels from gel shift assays comparing linear diubiquitin cleavage between the USP11 catalytic core and the USP11 ^D886G^ mutant with densitometric analysis shown below (error = SEM; n = 3). *C*, densitometric analyses of Lys^63^-linked di-Ub chain and Ub-GG-Ub chain cleavage assays (error = SEM; n = 3). *D*, kinetic data for USP11-D1D2 in comparison with USP11-D1D2 BL2 region D886G and R885G mutants (error = SD; n = 3). BL, blocking loop; ITC, isothermal titration calorimetry; USP, ubiquitin-specific protease.

Binding of the product mono-ubiquitin to USP11-D1D2 revealed an exothermic binding behavior and a K_D_ of ∼3.5 μM compared to 1.1 μM for USP11-D1D2 ^R885G^ and ∼2 μM for USP11-D1D2 ^H399Q^, respectively, showing that USP11-D1D2 ^R885G^ displayed slightly higher affinity for mono-ubiquitin (Fig. 4*B*). The USP11 catalytic triad mutant C318S showed a higher affinity for ubiquitin than WT, an observation that has been reported for other USPs (19, 28) (Fig. 4*B*). USP11-D1D2 ^C318S^ interacted with high affinity with both mono-Ub and substrate Ub-GGG but a ∼50-fold reduction in affinity was observed for the linear di-Ub substrate (Figs. 4*B* and 5*A* and S6). We also investigated USP11-D1D2 ^C318S^ binding to Ub-M, a maximally truncated linear di-Ub substrate with only the first methionine of the proximal ubiquitin moiety present (Fig. 1*D*). Binding assays with Ub-M unexpectedly still displayed a K_D_ that was significantly higher than Ub-GGG as well as Ub-G. Ub-G is equivalent to Ub-M, except for lacking the methionine side chain (Figs. 1*D* and 5*A*). This showed that the active site mutant USP11-D1D2 ^C318S^ binds considerably less tightly to linear di-Ub and Ub-M compared to Ub-GGG, Ub-G, or the reaction product mono-Ub. The data suggests that the presence of the start methionine of the proximal ubiquitin moiety in linear diubiquitin (the P1′ residue) is a key contributor to the significant reduction in the affinity observed for this substrate.

### An aspartate in the BL2 region modulates USP11’s substrate selectivity

USP11 is known to have a preference for Lys^6^- and Lys^63^-linked ubiquitin chains, whereas USP15 is more promiscuous. For example, linear diubiquitin is a very poor substrate for USP11 (15, 22, 23) and to a lesser degree USP4 (24, 29), but is readily cleaved by USP15.

**Figure 6:**
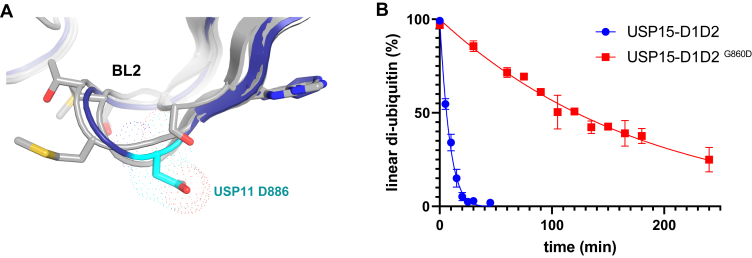
**Location of “gatekeeper residue” position in blocking loop 2 and impact of mutation on linear diubiquitin cleavage in USP15.***A*, closeup view of the USP11 Asp^886^ BL2 region (in *blue*) in a superposition with USPs that are known to readily cleave linear diubiquitin, namely CYLD (PDB ID: 2vhf, (62)), USP2 (PDB ID: 2hd5, (30)), and USP15 (PDB ID: 6gh9, (29)). *B*, densitometric analyses of linear di-Ub chain cleavage assays (error = SEM; n = 3) comparing USP15-D1D2 *versus* USP15-D1D2 ^G860D^. USP, ubiquitin-specific protease.

**Figure 7:**
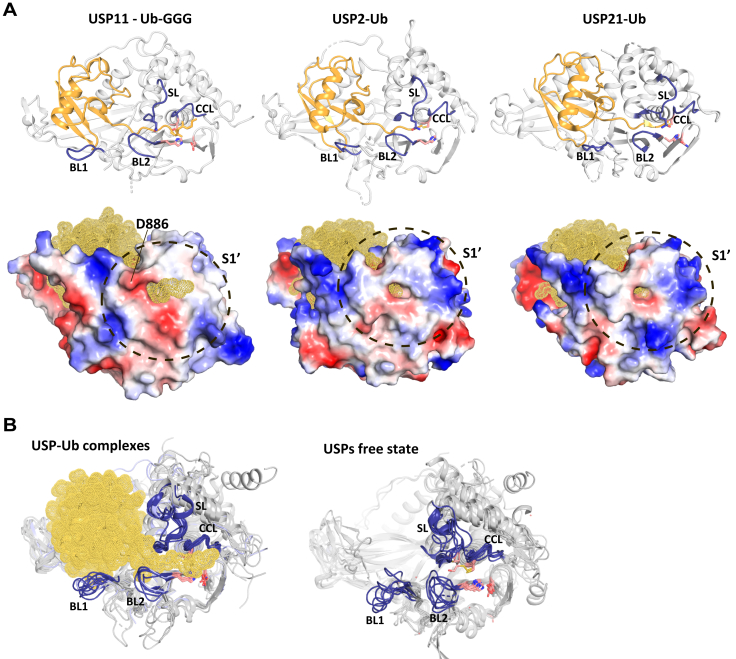
**Impact of distal ubiquitin binding on USP structures.***A*, comparison of USP11 to the closest available USP–Ub complex structures of USP2 (PDB ID: 2ibi) and USP21 (PDB ID: 3i3t). *Top*: cartoon diagram of USP catalytic domains in *gray* with active site loops shown in *blue* and Ub-GGG or ubiquitin, respectively in *light orange*. *Bottom*: electrostatic surface representations of same enzyme as above displaying the S1′ site and active site cleft channel with Ub-GGG transiting in the complex structure of USP11 or the product ubiquitin seen through the channel in *orange mesh representation* for USP2 and USP21. *B*, *left*: superposition of catalytic core structures in the presence of a Ub species in the S1 pocket: USP2 (PDB ID: 2hd5, (30)), USP7 (PDB ID: 1nbf, (63)), USP11, USP14 (PDB ID: 2ayo, (64)), USP21 (PDB ID: 2y5b, (31)), USP30 (PDB ID: 5ohk, (32)), and USP46 (PDB ID: 5cvo, (65)). CCL, SL, BL1, and BL2 regions are highlighted in *blue* and only Ub-GGG is shown depicted in *light orange*. *Right*: superposition of USP catalytic core structures in the absence of a ubiquitin species in the S1-binding pocket (free states): USP4 (PDB ID: 2y6e, (18)), USP7 (PDB ID: 2f1z, (66)), USP8 (PDB ID: 2gfo, (67)), USP9X (PDB ID: 5wch, (68)), USP12 (PDB ID: 5k16, (37)), USP14 (PDB ID: 2ayn, (64)), and USP15 (PDB ID: 6gha, (19)). Active sites loops are depicted in *blue* and the catalytic triad residues are shown in *light pink.* USP, ubiquitin-specific protease; Ub-GGG, ubiquitin-triple-gly.

The BL2 region forms part of the S1′ binding site and is not well conserved between the paralogs. In the crystal structure, aspartate residue Asp^886^ is central to the USP11 S1′ region’s charge and shape (Figs. 4*A*, 6*A*, and 7*A*). The conformation of the Asp^886^ side chain is defined by forming hydrogen-bonding interactions with the side chain of Gln^398^ in the SL. Asp^886^ is thus involved in closing the substrate glycine tail binding channel by linking the SL and BL2 loops in the Michaelis complex and protrudes from the surface (Fig. 7*A*). We therefore hypothesized Asp^886^ may contribute to USP11’s ability to discriminate against linear diubiquitin. To test this, we generated variant USP11-D1D2 ^D886G^, which is equivalent to residue Gly^860^ in USP15. USP11-D1D2 ^(C318S/D886G)^ bound linear diubiquitin and Ub-M with a higher affinity than when Asp^886^ was present confirming that Asp^886^ contributes to substrate interactions (Figs. 5*A* and S6). To assess the impact of this residue on the catalytic activity, we conducted enzymatic assays monitoring the gel-shift upon linear diubiquitin substrate cleavage for the WT *versus* the USP11-D1D2 ^D886G^ mutant. The data showed that the USP11-D1D2 ^D886G^ mutant was significantly more active against linear diubiquitin compared to the WT protein (Fig. 5*B*). Cleavage assays with Lys^63^-linked diubiquitin (Lys^63^-di-Ub) also displayed differences between WT and USP11-D1D2 ^D886G^ but with the opposite effect to linear di-Ub (Fig. 5*C*). In contrast, gel-shift assays with a linear di-Ub substrate engineered with an additional two glycine residues at the C terminus of the distal ubiquitin moiety (Ub-GG-Ub; Fig. 1*D*) resulted in rapid cleavage of the substrate irrespective of the D886G mutation. These results further indicate that the nature of the residue in the P1′ position of the substrate and its environment are important (Figs. 5*C* and S7). Enzymatic assays using minimal substrate ubiquitin-AMC displayed kinetic parameters consistent with slightly lower activity (Fig. 5*D*). This shows that USP11 with the D886G mutation despite displaying a slightly lower activity than WT using ubiquitin-7-amino-4-methylcoumarin as a substrate is significantly more active against linear di-Ub. The same is not observed for Lys^63^-di-Ub or Ub-GG-Ub. We next investigated the reciprocal G860D mutation in paralog USP15, which contains a glycine at the equivalent position to USP11 Asp^886^ (Figs. 3*A* and 6*A*). The results of the gel shift assay showed that the mutation has a considerable impact on USP15-D1D2’s rate of cleaving linear di-Ub (Figs. 6*B* and S7). Hence, this data reveals that Asp^886^ located in the BL2 loop of USP11 contributes to selective catalysis.

### Structural comparisons of the USP11–Ub-GGG complex with other USPs

**Figure 8:**
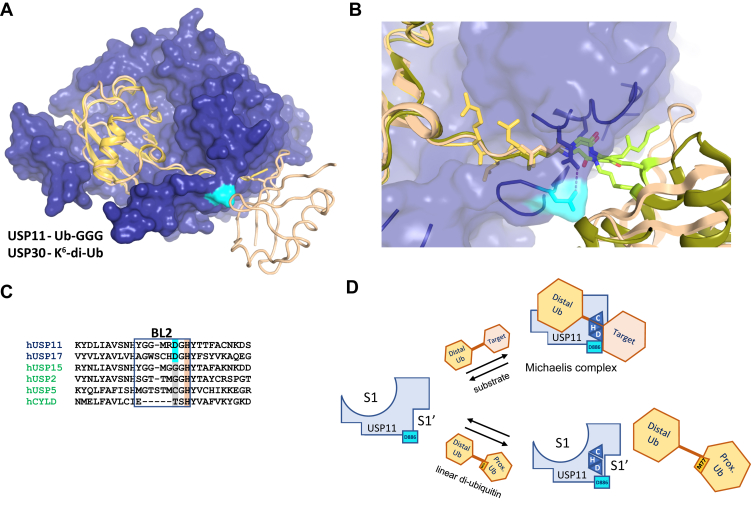
**Ub-GGG as a substrate mimetic and the position of the “gatekeeper” residue.***A*, *blue surface representation* of USP11-D1D2 with Ub-GGG in *yellow cartoon representation* superimposed onto the USP30 (PDB ID: 5ohp, (32)) structure in complex with a Lys^6^-linked diubiquitin substrate (*wheat color*) highlighting that the triple glycine in Ub-GGG locates to the S1′ site. The location of BL2 USP11 Asp^886^ is highlighted in *cyan* and is with minor loop rearrangements compatible with linear Lys^6^-linked di-ub binding to USP11 in this orientation. *B*, USP11 transparent surface representation in complex with Ub-GGG (*yellow*) with the location of trapped substrates of Lys^6^-linked di-Ub (*wheat color*) as seen in complex with USP30 (PDB ID: 5ohp) and Ub-FANCD2 (*olive color*) as seen in complex with USP1 (PDB ID: 7ay1, (34)) superimposed. The position of the BL2 and CCL is shown and Asp^886^ highlighted in *cyan*. Scissile bond residues are highlighted in *lime*. *C*, sequence alignment of the BL2 region in USPs for which it is known that linear diubiquitin is either a very poor substrate (*blue*) or a good substrate (*green*) (29). The position of BL2 Asp^886^ is highlighted in *cyan*. *D*, schematic depiction of the location of residues Asp^886^ in the USP11 S1′ site as well as Met^77^ in linear diubiquitin that were found to influence USP11 cleavage rates for linear diubiquitin. BL, blocking loop; CCL, catalytic cleft loop; USP, ubiquitin-specific protease; Ub-GGG, ubiquitin-triple-gly.

The phylogenetically closest USPs to USP11 for which structures are known apart from paralogs USP15 and USP4 are USP2, USP8, and USP21. No substrate trapped complexes are available for these USPs. Product bound structures of ubiquitin in the S1 site of USP2 (PDB ID: 2hd5; (30)) and USP21 (PDB ID: 2y5b; (31)) generally superimpose well with the USP11–substrate complex (43.0 % seq. id.; RMSD 0.97 Å over 308 aa and 41.1%; RMSD 1.2 Å over 302 aa with USP11, respectively). This includes the CCL, SL, and BL2 regions despite differences in the sequences. In contrast, the BL1 region adopts different conformations in the structures (Fig. 7, *A* and *B*) and USP11 residues Tyr^833^ and Arg^838^ that interact with the distal ubiquitin moiety are not conserved. Very few USP structures where substrates have been trapped are known to date and include USP30, USP22, and USP1–UAF1 complexes with Lys^6^-linked diubiquitin (32), mono-ubiquitinated H2B (33), and mono-ubiquitinated FANCI − FANCD2 (34), respectively. Amongst these, USP30 (selective for Lys^6^-linked poly-Ub, PDB ID: 5ohp) is the closest available USP catalytic core structure to USP11. Moreover, Lys^6^-linked Ub chains are good substrates for USP11 (15). A superposition of the USP30 structure in complex with Lys^6^-linked di-Ub and USP11 in complex with Ub-GGG shows good agreement (30.6% seq. id. and RMSD of 1.6 Å over 268 aa) despite differences in the sequences. The triple-gly tail in the Ub-GGG substrate aligns well with the Lys^6^-linked di-Ub lysine side chain and no major clashes of Lys^6^-linked di-Ub in this orientation with the USP11 catalytic core are apparent (Fig. 8*A*). Overall, these comparisons show that there are significant similarities in distal ubiquitin binding to the S1 site between closely related USPs and that the extended ubiquitin tail successfully mimics the lysine side chain in an isopeptide bond of a substrate. It also shows that the USP11 structure in complex with the substrate mimetic Ub-GGG captures the characteristics of a fully formed S1′ site.

## Discussion

There are 56 USPs encoded in the human genome (35), but only for about a quarter structural information on the protease domain is available. Here, we added the structure of USP11 to the knowledge base. In contrast to the structures of the paralogs USP4 (18) and USP15 (19, 20), USP11 has been trapped with a novel engineered USP substrate developed in this study. The Michaelis complex structure of USP11 reveals the characteristics of the active conformation, while USP4 and USP15 structures (18, 19) adopt “closed hand” conformations that can only accommodate ubiquitin upon “opening up”. For USP15, a substrate-assisted mechanism with a reconfiguration of the catalytic triad has been proposed (19). Whether USP11 also displays an inactive catalytic triad configuration in the free state remains to be determined. Irrespectively, the comparisons suggest that substantial conformational changes are required to accommodate the substrate. These likely include rearrangement or flexible-to-order transitions of the catalytic site loops SL, BL1, and BL2. Moreover, they suggest that both the S1 and S1′ sites only fully form upon substrate binding by induced fit. Structures in different conformational states (free and S1 pocket occupied) are available for only a few USPs and include USP7 (36), USP12 (37, 38) and USP28 (39, 40). In general, the CCL, SL, BL1, and BL2 active site loop regions display significant conformational differences between USPs in the free states. When the S1 pocket is occupied by a distal ubiquitin moiety, the CCL and BL2 regions adopt very similar conformations, whereas more variability is still observed in the BL1 and SL regions (Fig. 7*B*). Sigmoidal kinetic behavior suggests that conformational changes are a key regulatory mechanism also for USP11. Very little is known about how USPs specifically recognize their respective substrates with a few exceptions (32, 33, 34, 41). Here, a novel substrate for deubiquitination was developed by constructing ubiquitin with three additional glycines (Ub-GGG) to mimic the lysine side chain. We show that Ub-GGG is a suitable substrate mimetic. Ub-GGG successfully mirrors the conformation of substrates harboring an isopeptide bond around the scissile bond (Fig. 8, *A* and *B*). This is consistent with the similarities of the extended nature of consecutive glycine residues and a lysine’s side chain.

The molecular basis of substrate selectivity is poorly understood for the majority of USPs. The S1′ site in the protease domain is assumed to play an important role in substrate recognition. Most USPs are considered promiscuous when it comes to differently linked poly-ubiquitin chains (1). However, amongst 52 active USPs, only USP2, USP5, CYLD, and USP15 display high activity toward linear di-Ub (29). Other USPs either show low activity or are largely inactive toward linear diubiquitin (29) (Fig. 8*C*). The difference in substrate selectivity between USP11 and the paralog USP15 despite significant sequence and structural similarities is especially intriguing. We noted that a position in the BL2 prior to the catalytic histidine may be of importance. The glycine prior to the catalytic histidine is almost absolutely conserved amongst USPs. This glycine is likely required for allowing the conformational changes to close the ubiquitin tail binding channel *via* an interaction with a glutamine from the SL region upon substrate binding. The position adjacent to this conserved glycine is more variable. USP11 has an atypical aspartate (Asp^886^) at this position in the BL2 (Figs. 6*A* and 8*C*). In the structure, the side chain of Asp^886^ is a distinctive feature of the S1′ site (Figs. 4*A* and 8*B*). Only USP17 isozymes, for which linear di-Ub is also a very poor substrate (42) have an Asp or Asn at this position. Comparison of USP11 and the paralog USP15 and their respective mutants showed that the presence or absence of an Asp in this “gatekeeper” position influences their ability to efficiently cleave linear diubiquitin. It is likely that charge-repulsion and/or steric reasons result in negative selection and the different reaction rates observed (Figs. 6*A* and 7*A*, schematic in Fig. 8*D*), although other factors such as different conformational dynamics and the rate of product release could also contribute. It is tempting to speculate that the nature of the residue in this position may also influence substrate selectivity in other USPs, but this remains to be determined. For example, we noted that USP2, another promiscuous USP similar to USP15, also has a glycine in this position (Figs. 7*A* and 8*C*).

The most defining characteristic of linear diubiquitin is that the C-terminal glycine of the distal ubiquitin is conjugated to the N-terminal methionine (the P1′ position of the substrate) of the proximal ubiquitin *via* a peptide bond, as opposed to conjugation to one of the seven internal lysine side chains *via* isopeptide bonds in different linkage-type poly-ubiquitin chains (Fig. 1*D*). The presence of the Met^1^ residue from the proximal ubiquitin moiety alone (as in linear diubiquitin) already causes a reduction in affinity for USP11, compared to Ub-G. This suggests that the Met^1^ residue of the proximal ubiquitin moiety is involved in causing linear diubiquitin to be a poor substrate for USP11, but other residues will also contribute. This is in line with structural models of USP11-D1D2 in complex with Ub-M from AlphaFold (43), which show Asp^886^ in close proximity to Met^77^ (equivalent to Met^1^ from the proximal ubiquitin moiety; Fig. S8).

For USP30, Ser^477^ in the S1′ site was identified as a substrate selectivity marker residue to explain Lys^6^-linkage preference (32). However, this position is occupied by an aspartate (USP11 Asp^905^) in USP11, USP4, and USP15, so does not account for paralogue specific differences in this subfamily of USPs. Individual USPs have evolved in different ways, and we are only just beginning to understand the molecular determinants for substrate selectivity amongst this family of cysteine proteases. For example, ancillary domains can also contribute to USP selectivity for certain substrates with different substrate-binding sites having been identified in USP11 (5, 44) and other USPs such as USP1 (45) and USP7 (46).

In this study a crystallizable stable USP11 catalytic domain construct was designed by use of a novel insertion tag. N-terminal fusion tags for carrier-driven crystallization have been used in isolated cases including maltose-binding protein (47), the macro domain (48) and thioredoxin (49). These tags can improve expression and solubility issues in addition to providing extra crystal contacts but suffer typically from increasing the flexibility, which can hinder crystallization. Loop insertion tags on the other hand do not suffer this disadvantage to the same extent. To our knowledge these have only been used in connection with solving the structures of membrane proteins, probably because the prediction of loop regions is more straight forward. A prominent example is T4 lysozyme, which was inserted to determine the structure of the human β2 adrenergic receptor (50) or the structure of the neurokinin 1 receptor where a disordered loop was replaced with a thermostable *Pyrococcus abysii* glycogen synthase domain (51). Here, we identified RDFrzS as a novel insertion tag by systematically mining the PDB database for suitable candidate proteins and proved its suitability as a crystallization tag for the case of the USP11 catalytic domain. Given that flexible large loops and insertions are common in many proteins and can be ever better predicted using *ab initio* folding and homology modeling methods (43, 52), this approach will be applicable to other targets that evaded crystallization to date.

## Experimental procedures

### Data base mining for the identification of the RDFrzS tag

The PDB was screened for candidate proteins or domains with properties that may aid solubility and crystallization efficiency and that are compatible with being inserted into loop regions. This was followed by visual inspection in PyMOL (PyMOL | pymol.org) and criteria included (i) size <400 amino acids, (ii) no disulphide bonds, (iii) no ligands, (iv) X-ray diffraction to < 1.6 Å resolution, (v) location of C and N termini no further than 10 Å apart, and (vi) globularity. Furthermore, factors such as concentration at which crystals were obtained, surface charge distribution, and B-factors were considered. The N-terminal RD of social motility protein FrzS from *M. xanthus* (RDFrzS; PDB ID: 2gkg (26)) was finally selected as a candidate insertion tag. The only other insertion tag tested was the structural 50S ribosomal protein L30e E90A variant from *Thermococcus celer* with PDB code 1w41 (53) (template plasmid kindly provided by Kaming Lee and Kam Bo Wong, The Chinese University of Hong Kong), but a USP11-L30e fusion construct did not yield any crystals.

### Constructs, cloning, and mutagenesis

The constructs for USP11-D1D2, mono-Ub, and linear di-Ub have previously been described (15, 19). Throughout this manuscript USP11 residue numbering for isoform UniProt P51784 is used. The DNA sequence for RDFrzS (RD of *M. xanthus* social motility protein, residues 3–123) was ordered from GenScript with SER mutations K94A and K96S. The fusion construct of the USP11 catalytic core with the RDFrzS tag insertion (USP11-D1D2_ins_) was generated by restriction-free cloning in pET26b by replacing the ASTSK linker between the D1 and D2 domains (15) with the RDFrzS insertion tag sequence using the following primers fw: CCTTCTGCTACCTCAGTGTTCCACTGCCGGGTGCGAAAAAAATTCTGATTGTGGAAAG,rv: CTCAATGCACTCCTGCAGCCGCACCGGCGGAAAGCCAATCAGCGCG). An active site mutant and three loop mutants were generated using the Quick-Change mutagenesis protocol (C318S: Fw: CAATCTGGGCAACACGTCCTTCATGAACTCGGCCCTG, Rv: CAGGGCCGAGTTCATGAAGGACGTGTTGCCCAGATTG; H399Q: Fw: GGCTACCAGCAGCAAGACTCTCAGGAGCT, Rv: AGCTCCTGAGAGTCTTGCTGCTGGTAGCC; R885G: Fw: CCATTATGGGGGCATGGGTGATGGACACTACAC, Rv: GTGTAGTGTCCATCACCCATGCCCCCATAATGG) The active site mutant USP11-D1D2_ins_^C318S^ was generated using the following primers: Fw: CAATCTGGGCAACACGTCCTTCATGAACTCGGCCCTG, Rv: CAGGGCCGAGTTCATGAAGGACGTGTTGCCCAGATTG). The active site mutant USP11-D1D2 ^D886G^, as well as the double mutant USP11-D1D2 ^(C318S/D886G)^ were generated using site-directed mutagenesis using the Quick-Change mutagenesis protocol based on the USP11-D1D2 and USP11-D1D2 ^C318S^ templates (Fw: CATTATGGGGGCATGCGTGGTGGACACTACACAACATTTGC, Rv: GCAAATGTTGTGTAGTGTCCACCACGCATGCCCCCATAATG). The sequence for Ub-GGG (ubiquitin residues 1–76 extended by three glycines) was cloned into pCDFDuet-1 using NdeI and KpnI restriction endonucleases with primers Fw: GGGAATTCCATATGCAGATCTTCGTGAAGACTC and Rv: CGGGGTACCTCAACCACCACCCCCACCTCTGAGACGGAG. Ub-G was then generated by introducing a stop codon. Ub-GG-Ub was obtained as a synthetic gene (GenScript) and was cloned into pCDFDuet-1. USP15-D1D2 ^G860D^ was generated from a construct previously described (19).

### Protein expression and purification

All proteins including USP11-D1D2, mutants C318S, R885G, D886G, (C318S/D886G), and H399Q, USP11-D1D2_ins_, USP11-D1D2_ins_^C318S^, USP15-D1D2, and mutants as well as ubiquitin constructs were expressed in *Escherichia coli* BL21(DE3)-Codon Plus cells using 2YT broth growth medium. The complex of USP11-D1D2_ins_^C318S^ with substrate Ub-GGG was prepared from coexpression. All USP proteins and complexes were expressed and purified in the following way: After reaching an OD_60__0_ of ∼0.6, overexpression was induced with 0.5 mM IPTG and cultures grown for 16 h at 18 °C. For USP11 purifications, cells were harvested, resuspended in buffer A (50 mM Tris-Cl, pH 8, 300 mM NaCl, 20 mM imidazole, 10% glycerol), lysed by sonication, and clarified by centrifugation (24,000*g*, 1 h, 4 °C). The lysate was loaded onto a HiTrap chelating column (Cytiva) precharged with Ni^2+^ ions and protein eluted using an imidazole gradient (20–500 mM). The purest fractions were combined and concentrated, and proteins further purified by size-exclusion chromatography (SEC) on a Superdex 200 16/600 column (Cytiva) using SEC buffer: 50 mM Tris-Cl, pH 7.5, 150 mM NaCl, 1% (v/v) glycerol. Pure protein fractions were combined and concentrated.

### Crystallisation, data collection, and structure determination

Crystals of USP11-D1D2_ins_^C318S^ in complex with Ub-GGG were grown using 8.6 mg/ml protein in SEC buffer at 20 °C using the sitting drop vapor diffusion method in the following condition: 100 mM Tris-Bicine pH 8.5, 30 mM sodium nitrate, 30 mM sodium phosphate dibasic, 30 mM ammonium sulphate, 11.25% (v/v) MPD; 11.25% (v/v) PEG 1000; 11.25% (w/v) PEG 3350 with 5 mM CdCl_2_. Needle-like crystals were cryoprotected with an additional 30% glycerol added to the mother liquor. Data from crystals containing the USP11-D1D2_ins_^C318S^ in complex with Ub-GGG were collected at the Swiss Light Source beamline X06DA at a wavelength of 0.999995 Å and 100 K. Crystals belonged to space group P 2_1_ 2_1_ 2 with unit cell parameters of a = 94.32 Å, b = 186.10 Å, c = 75.76 Å. Data were processed using XDS (54) and AIMLESS (55), and the structure was solved by molecular replacement using coordinates of a truncated USP11 homology model generated by PHYRE2 (56), ubiquitin (PDB ID: 1ubi) and RDFrzS (PDB ID: 2gkg) as search models in PHASER (57). The crystal contained two USP11-D1D2_ins_^C318S^ molecules in complex with Ub-GGG in the ASU. Both copies of the USP11 catalytic core superimpose well with an RMSD of 0.29 Å (over 2995 Cα atoms). Data collection statistics are shown in Table 1.

### Model building, refinement, and validation

Model building was performed using COOT (cam.ac.uk) (58), while structure refinements were performed using REFMAC (59) and PHENIX (60). Model quality was assessed using MolProbity (61). For USP11-D1D2_ins_ in chain A, seven N- and eight C-terminal residues and for USP11-D1D2_ins_ in chain B eight N- and five C-terminal residues were not modeled due to flexibility. An overlay of the two copies in the ASU shows differences in the positioning of the RDFrzS tag but no significant differences between the two USP11 and Ub-GGG molecules. Additionally, all 79 residues of both Ub-GGG molecules were modeled. Density for glycerol, nitrate, phosphate, and cadmium ion ligands was observed in both copies of the complex. A disulphide bond links USP11 Cys^428^ residues from chain A and B, which is likely a crystallization artefact, as no dimerization was observed during purification. The absence of zinc was confirmed by a Zn K edge scan at DLS I24 compared to a scan for a crystal grown with ZnCl_2_ as an additive. In the final model 96.85% of residues are located in favored regions of the Ramachandran plot. Refinement statistics are shown in Table 1. Figures were generated using PyMOL (The PyMOL Molecular Graphics System, Version 2.0 Schrödinger, LLC).

### Enzymatic assays and ITC

Kinetic parameters or time courses for USP11-D1D2 or mutants C318S, R885G, H399Q, D886G, or USP11-D1D2_ins_ were determined using ubiquitin-AMC as fluorogenic substrate in buffer 50 mM Tris-Cl, pH 7.5, 150 mM NaCl, 1% (v/v) glycerol, 1 mM DTT. Deubiquitinase activity was measured in 384-well white plates in 30 μl reaction volume in triplicates using an EnVision 2104 multilabel plate reader at 25 °C (excitation: 355 nm, emission: 426/8 nm). Measurements were taken once per minute for 30 min. Data were fitted using nonlinear regression analysis in the GraphPad Prism software (Home - GraphPad) (allosteric sigmoidal model using the equation: Y = V_max_ x X^h^/(K_half_^h^ + X^h^)) to establish K_half_ and k_cat_ values. Gel-shift based linear diubiquitin cleavage assays were performed in 150 mM NaCl, 50 mM Tris–HCl, pH 7.5, 1 mM DTT, in triplicate at 25 °C. Reactions were started by adding linear di-Ub or Ub-GG-Ub to a final concentration of 5 μM to 400 nM of USP11-D1D2, USP11-D1D2 ^D886G^, USP15-D1D2, or USP15-D1D2 ^G860D^. Reactions were stopped by adding SDS-PAGE loading buffer and analyzed on 18% SDS-PAGE gels. Gels were stained with Colloidal Coomassie blue stain, scanned, and analyzed using ImageJ (https://imagej.nih.gov/ij/). Relative amounts of linear di-Ub, Lys63-di-Ub (UbiQ) or Ub-GG-Ub and mono-Ub for each time point were determined and plotted using GraphPad Prism. ITC data were measured using a PEAQ ITC instrument (Malvern). Different ubiquitin samples including mono-Ub, linear di-Ub, Ub-GGG, Ub-G, and Ub-M (200–500 μM) were titrated into USP11 protein samples (20–30 μM) in 50 mM Tris-Cl, pH 7.5, 150 mM NaCl, 1% (v/v) glycerol. Experimental settings were 25 °C, 180 s spacing of injections at a 750-rpm stirring speed. Analysis of the data was performed using the PEAQ ITC analysis software (MicroCal PEAQ-ITC Analysis Software v1.41 | Malvern Panalytical) (Malvern), fitting to a one-site binding model. Experiments were performed at least in triplicates.

## Data availability

The data that support the findings of this study are available from the corresponding authors upon reasonable request. Atomic coordinates and structure factors of the catalytic core of USP11 in complex with substrate Ub-GGG have been deposited in the Protein Data Bank (http://wwpdb.org/) with accession code 8OYP.

## Supporting information

This article contains supporting information (43).

## Conflict of interest

The authors declare that they have no conflicts of interest with the contents of this article.
