# Supplementary material for: Ubiquitin-specific protease 11 structure in complex with an engineered substrate mimetic reveals a molecular feature for deubiquitination selectivity
Source: J Biol Chem. 2023 Sep 28;299(11):105300. doi: 10.1016/j.jbc.2023.105300 (PMC10637973; doi:10.1016/j.jbc.2023.105300)
Supplement: Supporting information [file mmc1.pdf]

Supporting Information

Ubiquitin specific protease 11 structure in complex with an engineered substrate mimetic reveals a molecular feature for deubiquitination selectivity

Sigrun K. Maurer[1], Matthias P. Mayer[1], Stephanie J. Ward[1], Sana Boudjema[1], Mohamed Halawa[1], Jiatong Zhang[1], Simon G. Caulton[1,2], Jonas Emsley[1], and Ingrid Dreveny[1]*

[1]Biodiscovery Institute, School of Pharmacy, University of Nottingham, Nottingham NG7 2RD, United Kingdom.

[2]Present address: Institute for Microbiology and Infection, School of Biosciences, University of Birmingham, Birmingham B15 2TT, UK.

*To whom correspondence should be addressed: Ingrid Dreveny: Biodiscovery Institute, School of Pharmacy, University of Nottingham, Nottingham NG7 2RD, UK; ingrid.dreveny@nottingham.ac.uk; Tel.+44 1158468015

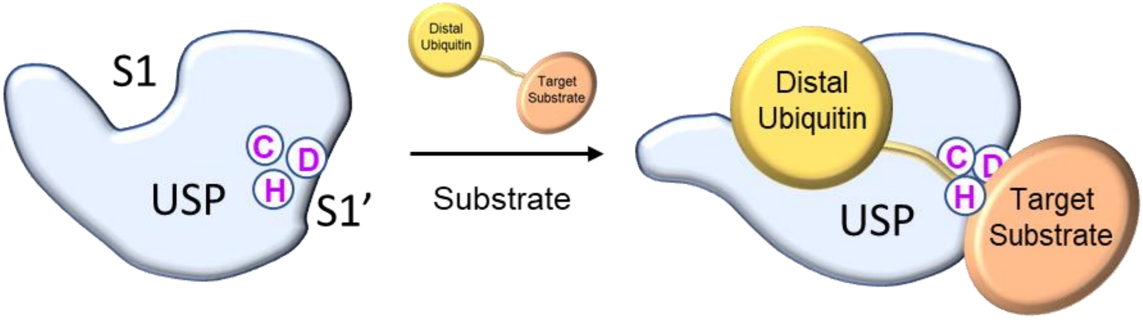

Fig. S1. Schematic representation of substrate binding to a ubiquitin specific protease catalytic domain. The USP catalytic domain composed of D1 and D2 sub-domains is depicted in light blue with the large S1 binding pocket for the distal ubiquitin moiety and much shallower S1’ binding site for the conjugated target substrate (which can be a second ubiquitin moiety and would in this case be termed proximal ubiquitin) labelled. Catalytic triad residues are represented in circles.

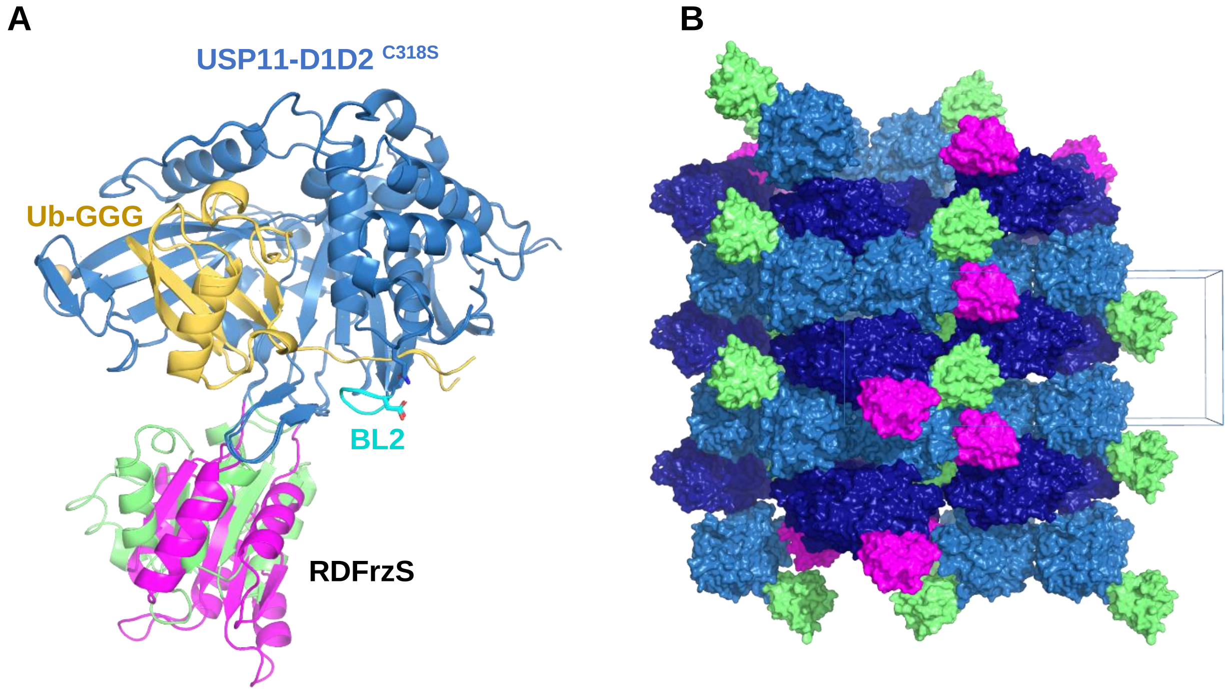

Fig. S2. Crystal packing and copies in asymmetric unit. ***A*** Superposition of the two copies of USP11-D1D2$_{ins}$ ^{C318S} structure in complex with Ub-GGG in the asymmetric unit as cartoon representation with USP11-D1D2 shown in blue and the blocking loop 2 region highlighted in cyan. Ub-GGG molecules are depicted in yellow and the RDFrzS insertion tags are highlighted in magenta and lime green, respectively. ***B*** Crystal packing of USP11-D1D2$_{ins}$ ^{C318S} structure in complex with Ub-GGG with molecules in surface representation. The two copies of the USP11-D1D2 ^{C318S} - Ub-GGG complex in the ASU are shown in light and dark blue, respectively. The RDFrzS insertion tags are colour coded as in ***A***. A unit cell is shown in blue.

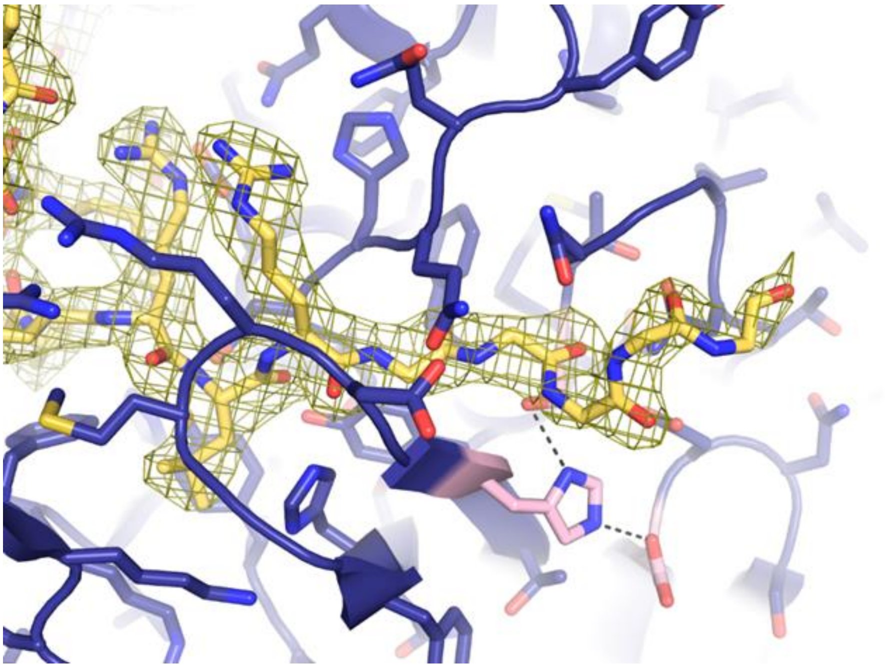

Fig. S3. Electron density of the extended ubiquitin tail region. Cartoon representation depicting the C-terminal residues of the minimal substrate Ub-GGG. USP11 is shown in blue, Ub-GGG in light orange and the catalytic triad residues with the C318S mutation are shown in light pink. Side chains are displayed as sticks and the sigmaA weighted 2Fobs − Fcalc electron density map for Ub-GGG is contoured at 1.05 σ.

A

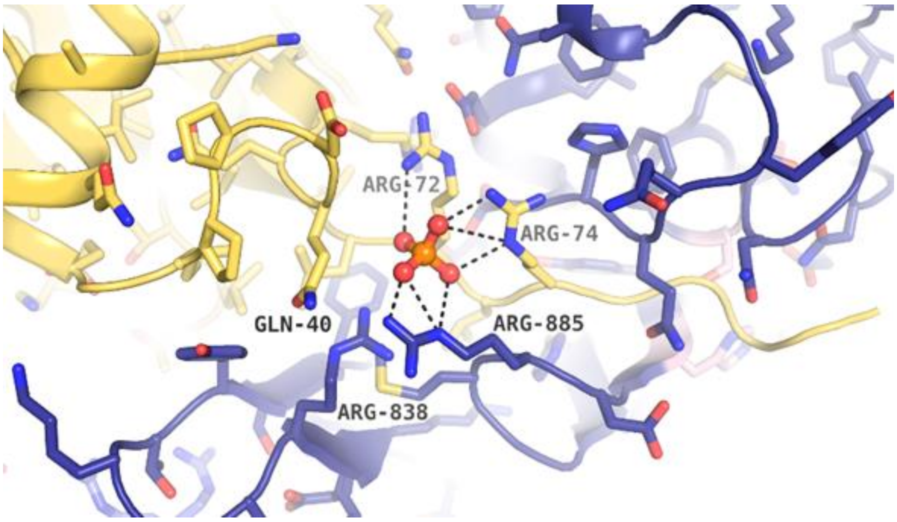

B

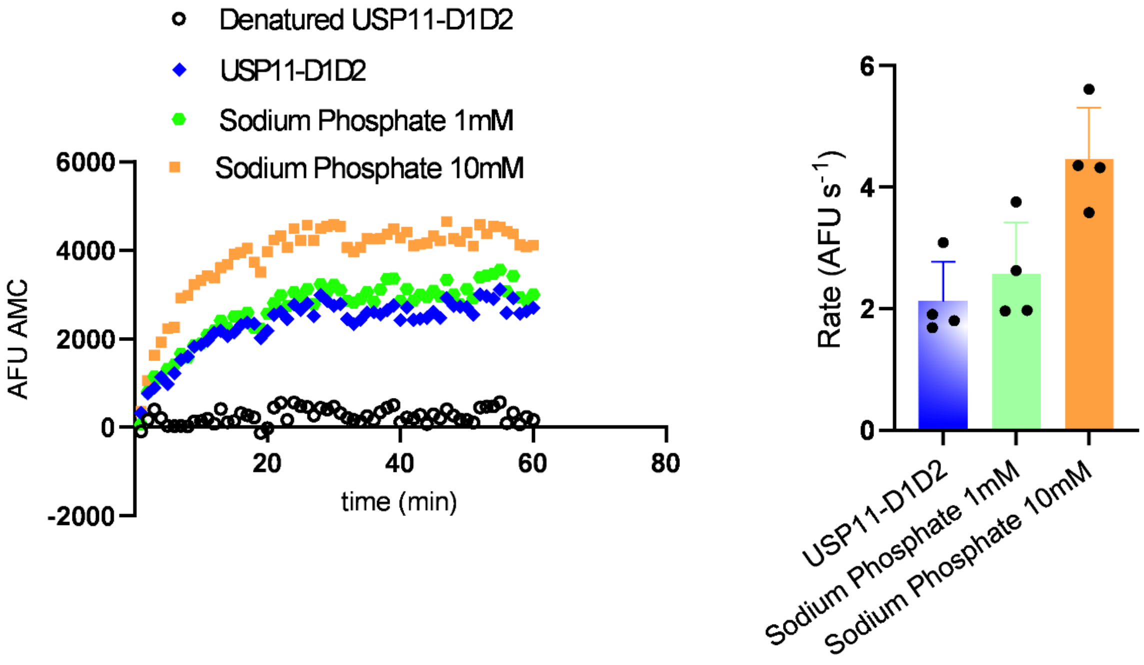

Fig. S4. Phosphate binding site in the structure of USP11-D1D2$_{ins}$ ^{C318S} in complex with Ub-GGG. ***A*** Detail of phosphate ion binding in the USP11 Ub-tail binding channel with engagement of the BL1 and BL2 regions (USP11 green, Ub-GGG yellow). ***B*** Progress curves of USP11-D1D2 (40 nM) - Ub-AMC (750 nM) cleavage reactions in the presence and absence of 1 mM (p-value 0.425) or 10 mM (p-value 0.0045) sodium phosphate with a bar chart (unpaired two-tailed t-test; n=4 independent experiments; error=SD) highlighting that at 10 mM sodium phosphate the rate is slightly enhanced.

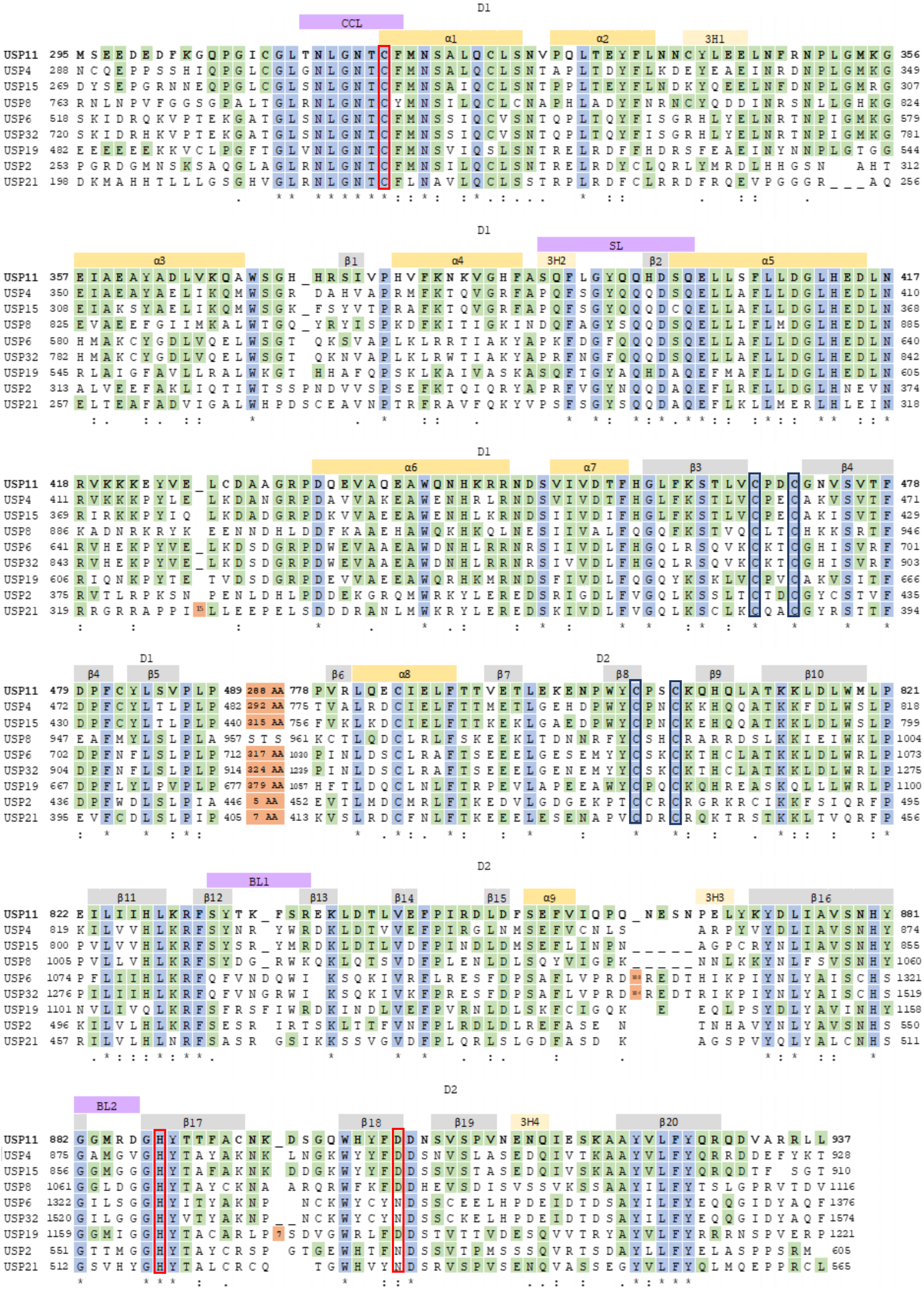
D1
CCL
α1
α2
3H1
USP11
USP4
USP15
USP8
USP6
USP32
USP19
USP2
USP21
α3
β1
α4
3H2
SL
β2
α5
α6
α7
β3
β4
β5
β6
α8
β7
D2
β8
β9
β10
BL1
β11
β12
β13
β14
β15
α9
3H3
β16
BL2
β17
β18
β19
3H4
β20

Fig. S5. Sequence alignment of selected USPs. Sequence alignment of the USP11 catalytic core with 8 closely related human USPs. The USP11 catalytic core sequence (295-489 = D1, and 778-937 = D2, UniProt P51784) is shown in alignment with the catalytic core sequences of USP4 (UniProt Q13107), USP15 (UniProt Q9Y4E8-2), USP8 UniProt (P40818), USP6 (UniProt P35125), USP32 (UniProt Q8NFA0), USP19 (UniProt O94966), USP2 (UniProt O75604) and USP21 (UniProt Q9UK80), alignment performed using Clustal Omega. Full sequence conservation between the proteins is highlighted in blue, partial sequence conservation compared to USP11 is marked in green. The following conservation markers of the Clustal Omega output are also shown: * = fully conserved; : = similar properties; . = weakly similar properties; blank = no conservation. The CCL, SL, BL1 and BL2 loop regions are indicated in purple. The catalytic triad and zinc finger residues are boxed in red and blue, respectively. Also indicated are the secondary structure elements (α-helix (yellow), 310-helix (light yellow), β-strand (grey)) of USP11 as seen in the USP11-D1D2 ^{C318S} substrate complex structure, assigned using the DSSP server (https://swift.cmbi.umcn.nl/gv/dssp/).

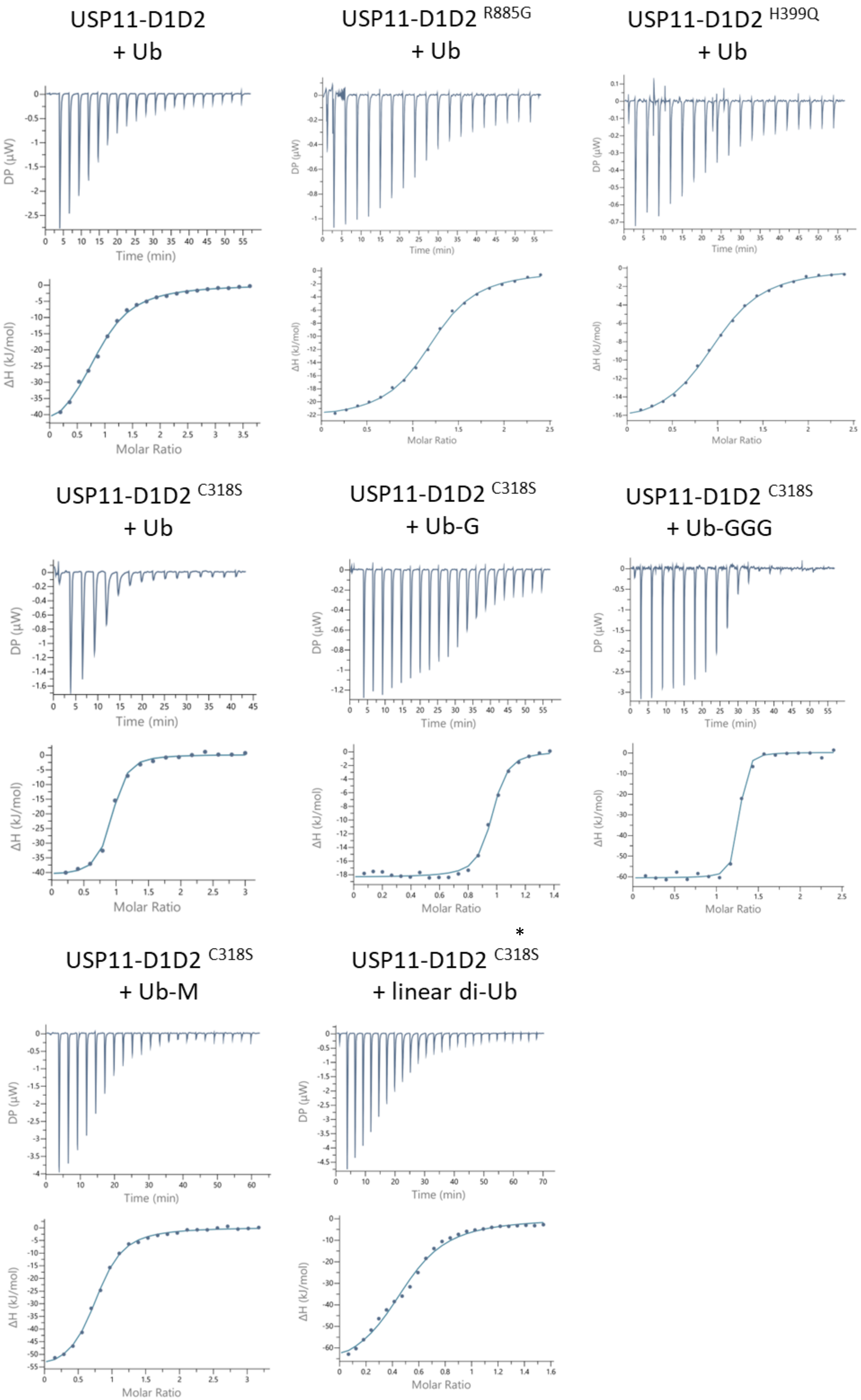
USP11-D1D2
+ Ub
DP (μW)
Time (min)
ΔH (kJ/mol)
Molar Ratio
USP11-D1D2 R885G
+ Ub
DP (μW)
Time (min)
ΔH (kJ/mol)
Molar Ratio
USP11-D1D2 H399Q
+ Ub
DP (μW)
Time (min)
ΔH (kJ/mol)
Molar Ratio
USP11-D1D2 C318S
+ Ub
DP (μW)
Time (min)
ΔH (kJ/mol)
Molar Ratio
USP11-D1D2 C318S
+ Ub-G
DP (μW)
Time (min)
ΔH (kJ/mol)
Molar Ratio
USP11-D1D2 C318S
+ Ub-GGG
DP (μW)
Time (min)
ΔH (kJ/mol)
Molar Ratio
USP11-D1D2 C318S
+ Ub-M
DP (μW)
Time (min)
ΔH (kJ/mol)
Molar Ratio
*
USP11-D1D2 C318S
+ linear di-Ub
DP (μW)
Time (min)
ΔH (kJ/mol)
Molar Ratio

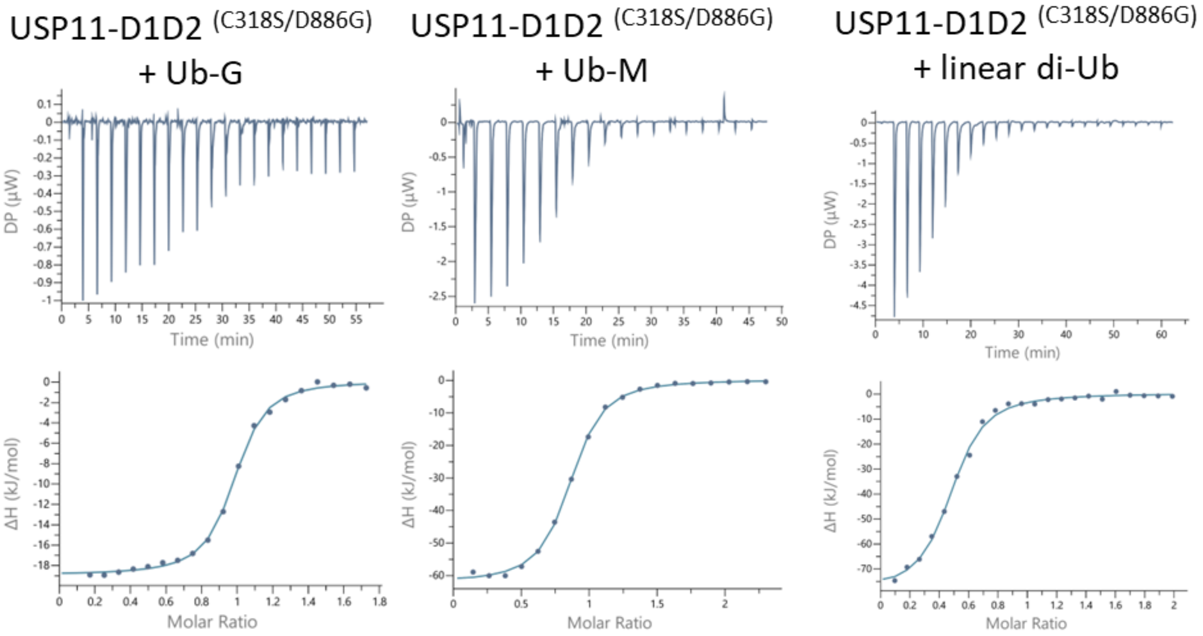

Fig. S6. Isothermal Titration Calorimetry data. Representative examples of ITC raw data and binding isotherms fitted to a one site binding model for USP11 catalytic cores and different substrates, or the product mono-ubiquitin measured at 25°C. * for the USP11-D1D2 ^{C318S} - linear di-Ub titration curve there are indications for two binding sites, but fitting does not converge using a two binding site model.

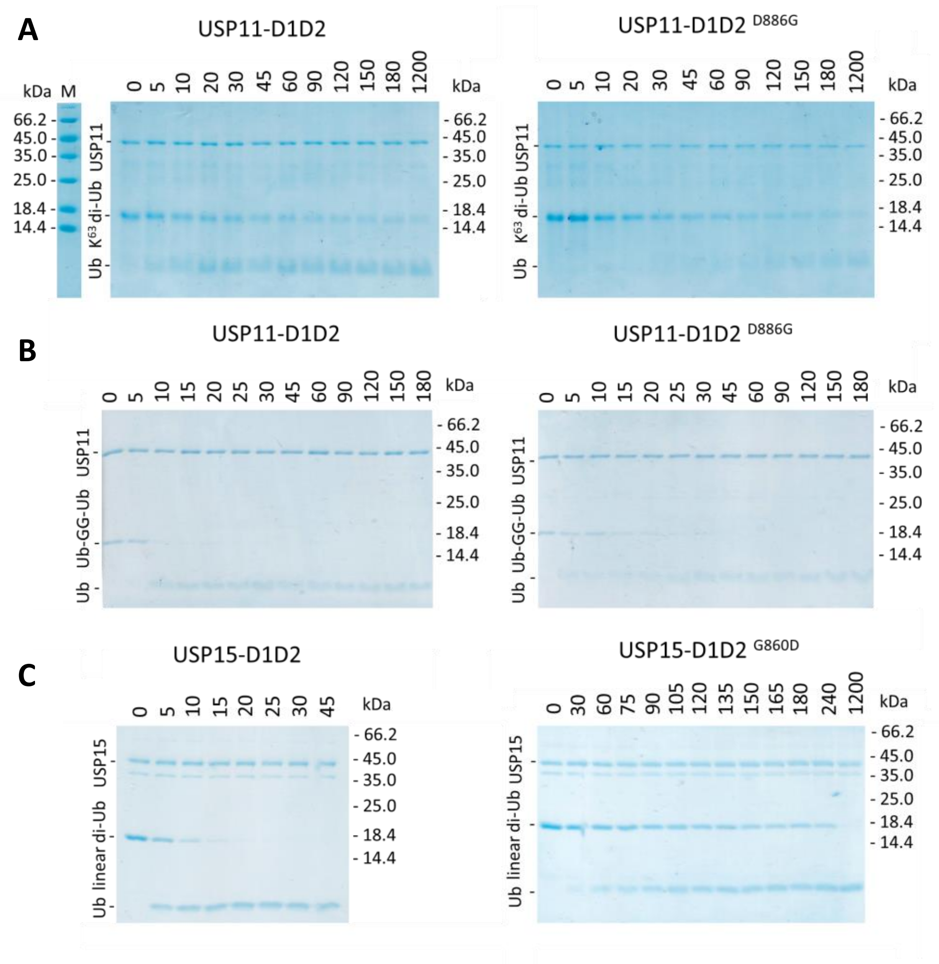

Fig. S7. **Substrate cleavage assays**. Representative examples of SDS-PAGE gels of cleavage assays for ***A, B*** USP11-D1D2 vs USP11-D1D2 ^{D886G} and ***C*** USP15-D1D2 vs USP15-D1D2 ^{G860D} catalytic cores with different substrates including Lys^{63}-linked di-Ub (***A***), Ub-GG-Ub (***B***) or linear di-Ub (***C***).

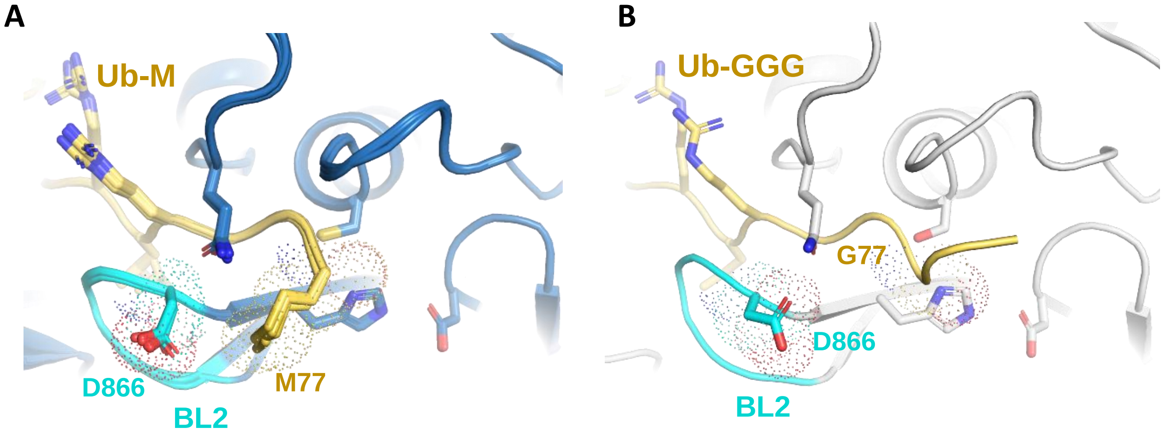

Fig. S8. **Comparison of the predicted USP11-D1D2 structural model in complex with Ub-M with the USP11-D1D2 - Ub-GGG crystal structure.** ***A*** Cartoon representation of the top five USP11-D1D2 models (blue) in complex with Ub-M (yellow) computed by AlphaFold (1). The BL2 region is highlighted in cyan and USP11 Asp^{866}, Gln^{398} and the catalytic triad residues are shown in stick representation as well as the C-terminal residues of Ub-M. In addition, USP11 Asp^{866} and Ub-M Met^{77} are shown in dots representation. USP11 Asp^{866} faces away from the Ub-M C-terminal methionine. ***B*** Crystal structure of USP11-D1D2 (grey) in complex with Ub-GGG (yellow) as reported here. Colour coding as well as residues shown in sticks are equivalent to (***A***). USP11 Asp^{866} and Ub-GGG Gly^{77} are shown as dots.

References

1. Jumper, J., Evans, R., Pritzel, A., Green, T., Figurnov, M., Ronneberger, O., Tunyasuvunakool, K., Bates, R., Zidek, A., Potapenko, A., Bridgland, A., Meyer, C., Kohl, S. A. A., Ballard, A. J., Cowie, A., Romera-Paredes, B., Nikolov, S., Jain, R., Adler, J., Back, T., Petersen, S., Reiman, D., Clancy, E., Zielinski, M., Steinegger, M., Pacholska, M., Berghammer, T., Bodenstein, S., Silver, D., Vinyals, O., Senior, A. W., Kavukcuoglu, K., Kohli, P., and Hassabis, D. (2021) Highly accurate protein structure prediction with AlphaFold. *Nature* **596**, 583-589
